# Evaluation of circulating sRAGE in osteoporosis according to BMI, adipokines and fracture risk: a pilot observational study

**DOI:** 10.1186/s12979-017-0097-0

**Published:** 2017-06-14

**Authors:** Emanuela Galliera, Monica Gioia Marazzi, Carmine Gazzaruso, Pietro Gallotti, Adriana Coppola, Tiziana Montalcini, Arturo Pujia, Massimiliano M. Corsi Romanelli

**Affiliations:** 1 Department of Biomedical, Surgical and Oral Science, Università degli Studi di Milano, Milan, Italy; 2 IRCCS Galeazzi Orthopaedic Institute, Milan, Italy; 3 Department of Biomedical Sciences for Health, Università degli Studi di Milano, Milan, Italy; 4 Internal Medicin, Diabetes, Vascular and Endocrine-Mtabolical Disease Unit and the Centre of Applied Clinical Research (Ce.R.C.A), Clinical Institute Betato Matteo, Vigevano, Italy; 5 Clinical Nutrition Unit, Department of Medical and Surgical Science, University Magna Grecia of Catanzaro, Catanzaro, Italy; 6 U.O.C SMEL-1 Patologia Clinica IRCCS Policlinico San Donato, San Donato, Milan, Italy

**Keywords:** Circulating RAGE, Glycation end products, Osteoporosis, Fracture risk

## Abstract

**Background:**

Osteoporosis is a systemic metabolic disease based on age-dependent imbalance between the rates of bone formation and bone resorption. Recent studies on the pathogenesis of this disease identified that bone remodelling impairment, at the base of osteoporotic bone fragility, could be related to protein glycation, in association to oxidative stress. The glycation reactions lead to the generation of glycation end products (AGEs) which, in turn, accumulates into bone, where they binds to the receptor for AGE (RAGE). The aim of this study is to investigate the potential role of circulating sRAGE in osteoporosis, in particular evaluating the correlation of sRAGE with the fracture risk, in association with bone mineral density, the fracture risk marker FGF23, and lipid metabolism.

**Results:**

Circulating level of soluble RAGE correlate with osteopenia and osteoporosis level. Serum sRAGE resulted clearly associated on the one hand to bone fragility and, on the other hand, with BMI and leptin. sRAGE is particularly informative because serum sRAGE is able to provide, as a single marker, information about both the aspects of osteoporotic disease, represented by bone fragility and lipid metabolism.

**Conclusions:**

The measure serum level of sRAGE could have a potential diagnostic role in the monitoring of osteoporosis progression, in particular in the evaluation of fracture risk, starting from the prevention and screening stage, to the osteopenic level to osteoporosis.

## Background

Osteoporosis is defined, according to 2001 NHI consensus conference, as a systemic disease characterized by loss of bone volume and resulting in a reduced skeletal integrity leading to a higher risk of bone fracture [1, 2]. Osteoporosis develops because of an age-dependent imbalance between the rates of bone formation and bone resorption [3] and is an emerging disease due to the increase of the population average age and its socioeconomic and public health impact is estimated to significantly increase in the next future. Due to the association with bone fragility and fracture risk, this disease interferes not only with the quality of life but also with life expectancy, in particular in elder people [4, 5] . The current recommendation for osteoporosis screening is the diagnosis by Bone Densitometry or DXA (Dual-energy X-ray absorptiometry) Scan, but even though this approach correlates with fracture risk and it can considered a good fracture predictor, many additional factors play a role in determining risk of fracture. While the first diagnosis of osteoporosis is based on bone mineral density (BMD), the evaluation of the individual fracture risk should consider all the clinical risk factors, ranging from age, sex, metabolic status and bone remodelling status [6, 7].

On the one hand, DXA scan gives the static measurement of bone density in specific bone sites, on the other hand, additional diagnostic tool, such as bone turnover biomarkers, reflects the dynamic changes of bone turnover in the whole skeletal [8–10]. Therefore, the comprehension of the biochemical mechanisms involved in the pathogenesis of osteoporosis could provide new diagnostic tool for improving the sensibility and specificity the prediction of fracture risk [7].

Osteoporosis is a systemic metabolic disease and recent studies on the pathogenesis of this disease identified that bone remodelling impairment, at the base of osteoporotic bone fragility, could be related to protein glycation, in association to oxidative stress [5]. The glycation reactions lead to the generation of glycation end products (AGEs) which, in turn, accumulates into bone, where they binds to the receptor for AGE (RAGE) [11]. The RAGE receptor belongs to the immunoglobulin superfamily of cell- surface molecules expressed in different cell types, including osteoclasts and osteoblasts. Upon binding AGEs, RAGE increase osteoclasts activity and decrease osteoblasts activity [12, 13], thus contributing to increase bone resorption and, ultimately, bone fragility [5, 14].

The negative action of AGEs-RAGE axis is compensated by the soluble form of RAGE receptor (sRAGE), which is a decoy receptor of AGEs . Being a soluble receptor, sRAGE binds AGEs but doesn’t lead to any signaling pathway, thus competing with the signaling, cell-bound RAGE receptor and, as a consequence, limiting the AGEs-RAGE axis action [15].

RAGE may be bound by many ligands which include advanced glycation endproducts (AGEs), certain mem-bers of the S100/calgranulin family, extracellular HMGB1-amphoterin, the integrin Mac-1, amyloid beta-peptide and amyloid fibrils. Acting as counter-receptor for leukocyte integrins RAGE may also have an important role in cell adhesion and clustering as well as recruitment of inflammatory cells [16, 17]. Other important ligands for RAGE may be glycosaminoglycans (including chondroitin sulfate, dermatan sulfate and heparan sulfate) which are frequently attached to proteoglycans on the surface of cancer cells and play an important role in the malignant transformation of the tumor and metastasis [17].

Being involved in the inflammatory response [18], RAGE ligand (s)/RAGE system, in particular the axes AGE-RAGE is involved in the pathogenesis of a variety of inflammatory disorders, ranging from diabetes to renal disease, sepsis and cardiovascular disease [11]. In particular, the circulating soluble form of the RAGE receptor (sRAGE) has been recently described as a marker of disease in different pathologies, ranging from cardiovascular disease to acute liver failure [19] and metabolic disorder in obesity and diabetes [5, 20], but the diagnostic role of sRAGE in osteoporosis has not been described so far.

The aim of this study is to investigate the potential role of circulating sRAGE in osteoporosis, in particular evaluating the correlation of sRAGE with the fracture risk, in association with bone mineral density e and the fracture risk marker FGF23 (Fibroblast Growth Factor 23), involved in bone mineral metabolism. Since osteoporosis pathogenesis is strictly related with the metabolic status [21, 22], the aim of this study is also to evaluate the role of sRAGE as marker of osteoporosis in correlation with BMI and the adipokines leptin, adiponectin and visfatin.

## Methods

### Patients

The study involves 84 postmenopausal female volunteers (mean age 53 ± 6 years old) enrolled in the region of Lombardia and Calabria, as described in our previous work (Montalcini et al. 2015 [23]). All participants were evaluated for familiarity of osteoporosis and past fractures, medication use, physical exercise and smoke habits. Post menopausal status was defined as FSH level higher than 40 IU/l or one year at least of absence of natural mense.

Exclusion criteria were: presence of diabetes or any metabolic condition affecting bone metabolism (kidney, thyroid, rheumatic and hematological disease, malignant tumors) taking drug, hormone therapy or vitamin D affecting bone metabolism. The patients in our study was not under bisphosphonates therapy or other anti-osteoporotic therapy.

Written informed consent was obtained from all participants included in the study. All procedures followed were in accordance with the Helsinki of Declaration of 1975, as revised in 2000 and 2008 and it was approved by both the Ethic Committee ASL Milan 2 and The University Hospital Mater Domini, Catanzaro (Italy).

Trial registration number: 2631, name of registry: Comitato Etico indipendente della ASL Milano due. URL: http://www.aslm2.it, date of registration: September 19, 2011. Date of enrolment of the first participant 1 October 2011.

### DXA assessment

BMD evaluation of lumbar spine and left femoral was assessed by DXA (Horologic QDR Inc., MA. USA). BMD was expressed as T -score (number of standard deviation from healthy young mean) and Z- score (number of standard deviations from healthy women of the same age) calculated on the basis of physiological reference values. On the basis of T-score value the patients was classified as having normal bone (T-score > −1), osteopenia (Tscore: −1 to −2.49) or osteoporosis (T-score ≤ − 2.5).

In vivo precision, calculated as repeated measurements on 30 women, was <1%.

### Anthropometric measurement

Body weight and BMI was measured as described in Montalcini et al. 2015 [23]. Briefly, body weight was measured with a calibrated scale before breakfast subtracting the weight of clothes. Height was measured by wall- mounted stadiometer and BMI was calculated as weight (kg)/(height (m)) ^2^. According to BMI values, patients were classified as obese (BMI > 30), overweight (BMI 25–30) or normal weight (BMI < 25).

### Glomerular filtration rate

Glomerular filtration rate was calculated for the whole population and for each RAGE quartile by CKD-EPI formula:

GFR = 141 * min(Scr/κ,1)^α^ * max(Scr/κ, 1)^-1.209^ * 0.993^Age^ * 1.018 [if female] * 1.159 [if black]Scr is serum creatinine (mg/dL), κ is 0.7 for females and 0.9 for males, α is −0.329 for females and −0.411 for males, min indicates the minimum of Scr/κ or 1, and max indicates the maximum of Scr/κ or 1.

### sRAGE, FGF23 and adipokines ELISA assay

Blood was sampled from all patients and sera were separated from whole blood after complete coagulation, by centrifugation at 3000 rpm for 10 min. Sera were stored at −70 °C until ELISA assay analyses.

Levels of soluble RAGE, C-Terminal FGF23, Leptin, Adiponectin and Visfatin in serum were determined by commercial assays, according to the manufacturers’ instructions (sRAGE: R&D Systems, Minneapolis, Minnesota, USA; C-Terminal FGF23: Imuunotopiscs, San Clemente, CA, USA; Leptin: Enzo Life Sciences, Farmingdale, New York, USA; Adiponectin and Visfatin: AdipoGen AG, Liestal, Switzerland).

For the sRAGE assay, the sensitivity was 4.44 pg/mL, and intra- and inter-assay coefficients of variation were 2.4% and 4.7%, respectively. For the FGF23 assay, the sensitivity was 1.5 RU /mL, and intra- and inter-assay coefficients of variation were2.4% and 4.7%, respectively. According to manufacturer (, Imuunotopiscs, San Clemente, CA, USA) 1RU roughly equates to 2 pg/mL. For the Leptin assay, the sensitivity was 23.4. pg/mL, and intra- and inter-assay coefficients of variation were 4.4% and 3.7%, respectively. For the Adiponectin assay, the sensitivity was 1 ng/mL, and intra- and inter-assay coefficients of variation were 3.3% and 2.75%, respectively. For the Visfatin assay, the sensitivity was 30.0 pg/mL, and intra- and inter-assay coefficients of variation were 2.3% and 4.6%, respectively.

### Statistical analysis

For all parameters, the normality of distribution of the three groups was verified by KS (Kolmogorov-Smirnov) normality and results are reported as mean ± standard deviation (SD). Statistical analysis was done using one-way ANOVA, *p* < 0.05 being considered significant and *p* < 0.001 highly significant. The correlation of sRAGE with LBMD (lumbar bone mineral density) and FBMD (femoral bone mineral density) (expressed as T-score and Z-score) and with FGF23 was calculated as Spearman correlation (r) calculating the 95% confidence interval. All statistical analysis was performed using PRISM 3.0 software.

## Results

### Glomerular filtration rate and sRAGE

Glomerular filtration rate was calculated by CKD-EPI formula for the whole population and for each RAGE quartile, in order to evaluate whether it could affect sRAGE levels in our patients . The mean eGFR calculated on the whole patient group was 88,71 ± 11,86 mL/min per 1.73 m^2^, while it resulted 85,05 ± 10,74, 89,45 ± 12,71, 88,91 ± 9,56, 84,85 ± 9,13 mL/min per 1.73 m^2^ in the first, second, third and fourth sRAGE quartiles, respectively. Therefore, no significative differences in eGFR were observed among sRAGe quartiles and compared to total mean eGFR.

### sRAGE and bone mineral density

A total of 12 subject present osteoporosis, while 32 had osteopenia and 40 normal bone density values, as assessed by T-score and Z- score.

**Fig. 1:**
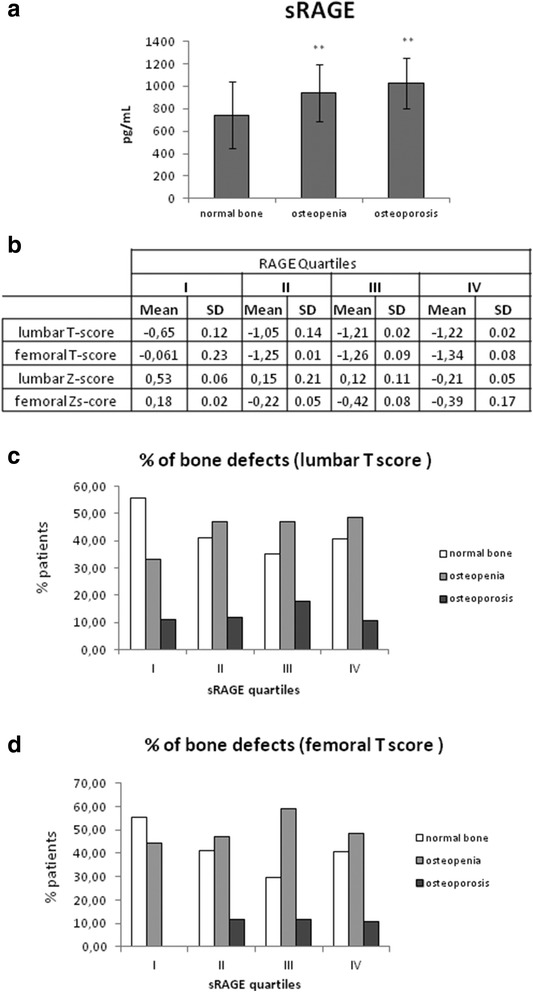
Serum sRAGE and bone density. Panel **a**: serum sRAGE (pg/mL) in patients displaying normal bone, osteopenia and osteoporosis, according to Bone Mineral Density valuesPanel **b** bone mineral density, expressed as lumbar and femoral T- and Z-score, according to sRAGE quartiles Panel **c** and **d**: percentage of patients in each sRAGE quartiles displaying normal bone, osteopenia and osteoporosis, according to Bone Mineral Density values, expressed as lumbar T-score (panel **c**), and femoral T-score (panel **d**)

The serum level of circulating s RAGE was measured in the three groups of patients (osteopenic, osteoporotic and normal bone, according to T-score values). sRAGE displays a significative increase in osteopenic patients (940.36 ± 206.79 pg/mL) and an even more significative increase in osteoporotic patients (1028,54 ± 223.83 pg/mL) compared to patients with normal bone (743.02 ± 204.91 pg/mL), (Fig. 1, panel a). The population was divided according to sRAGE quartiles, calculating in each quartiles the mean lumbar and femoral T-score and Z-score values (Fig. 1, panel b) and the amount of patients having osteopenia, osteoporosis and normal bone according to LMBD and FBMD T-score and T-score values ranges (Fig. 1 panel c and d, respectively). The first quartiles of sRAGE present no pathological T-score or Z-score, the second and third quartile present osteopenia according to T-score but normal bone according to Z-score, while the fourth quartile present a more severe osteopenia according to both T-score and Z-score mean value.. The first quartile of sRAGE showed the highest percentage of patients with normal bone and the lower percentage of osteopenia and osteoporosis according to lumbar T-score (Fig. 1, panel c and d). More significantly, classification based on Femoral T-score (panel D) not only confirmed the percentage of normal and osteopenic patients, but resulted in no cases of osteoporosis in the lowest quartile of sRAGE. The second, third and fourth quartile of sRAGE display very similar classification, having higher percentage of osteopenic patient and a lower percentage of normal bone compared to the first quartile. More interestingly, while osteoporotic percentage is nearly constant in the three upper quartiles according to femoral T-score, lumbar T-score classification underlined a little increase of osteoporosis percentage in the fourth quartile of sRAGE. These results indicates increase of osteoporosis percentage according to sRAGE levels.

### sRAGE and fracture risk

**Fig. 2:**
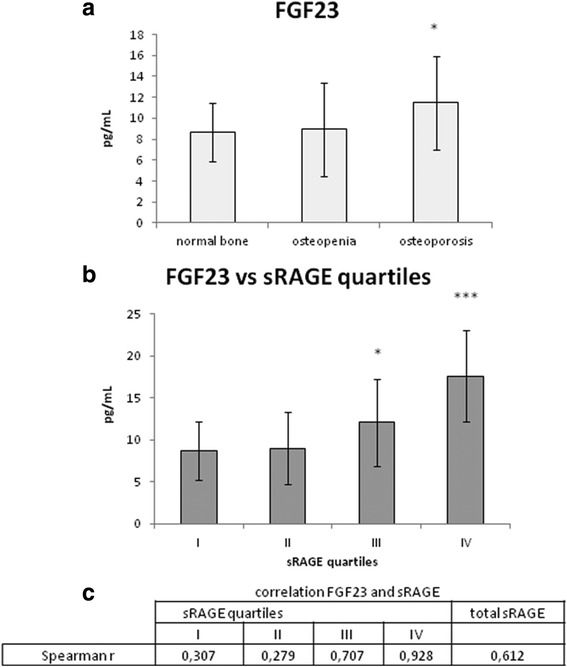
sRAGE and FGF23. Panel **a** serum FGF23 (pg/mL) in patients displaying normal bone, osteopenia and osteoporosis, according to Bone Mineral Density values Panel **b** serum FGF23 (pg/mL) in each sRAGE quartiles Panel **c** Spearman Correlation of serum FGF23 (pg/mL) with sRAGE as total level and sRAGE quartiles

There is significative trend of increase of FGF23 according to bone defect classification (Fig. 2 panel a) and sRAGE quartiles (panel B), in particular the first quartile displaying the lowest FGF23 value, below the cutoff of fracture risk in the first quartile and reaching the highest and very significative value in the highest quartile of sRAGE.

The Spearman correlation analysis (Panel C) did not find a strong correlation between serum FGF23 and the whole sRAGE values, while after the classification into sRAGE quartile an increasing correlation with serum FGF23 emerged, reaching high value in the third and even more in the fourth sRAGE quartile. These results indicates a correlation between sRAGE and fracture risck marker FGF23.

### sRAGE and BMI

**Fig. 3:**
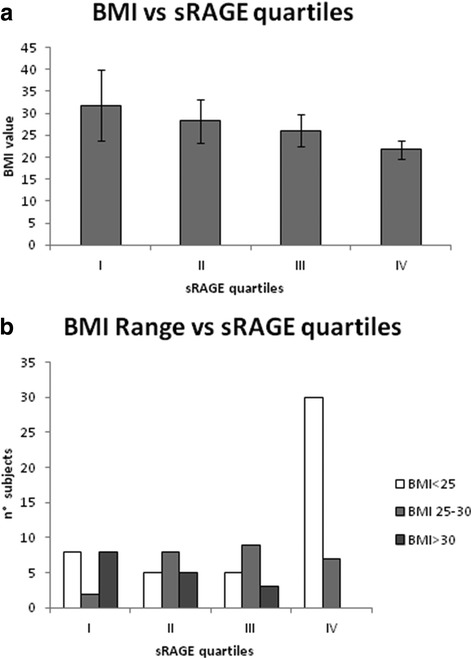
sRAGE and BMI. Panel **a** BMI (mean ± SD) in patients in each sRAGE quartiles Panel **b** BMI classification (<25 normal weight, 25–30 overweight, >30 obese) in each sRAGE quartiles serum FGF23 (pg/mL) in each sRAGE quartiles

The categorization into sRAGE quartiles was applied to analyze the metabolic status of the patients, evaluated by BMI calculation. There is a trend, even if not statistically significative, of decrease of BMI according to the increase of sRAGE (Fig. 3 panel a). In order to analyze this result in detail, we calculated the number of patients in each sRAGE quartile having normal (<25), overweight (25–30) or obese (>30) BMI (Fig. 3 panel b). According to sRAGE quartiles, there is a trend of decrease of obesity (BMI >30), reaching in the fourth sRAGE quartile the complete absence of obese subject and a very high prevalence of subjects with normal BMI. These results indicates a trend of decrease of obesity according to sRAGE levels.

### BMI and bone status

**Fig. 4:**
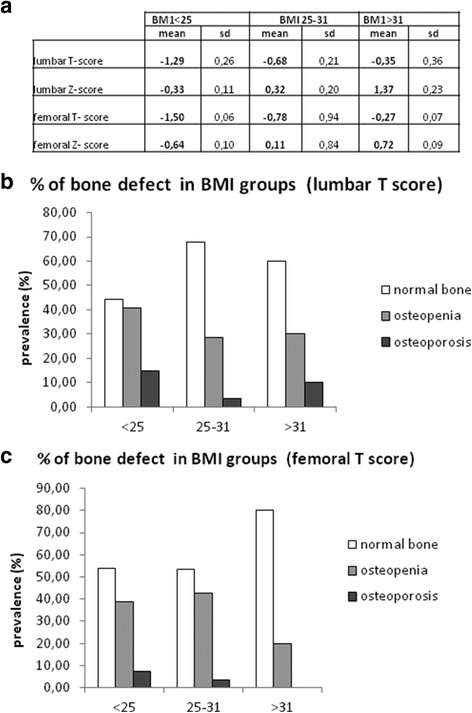
BMI and bone status. Panel **a** Bone Mineral Density values, expressed as lumbar and femoral T-and Z. core, according to BMI classification (<25 normal weight, 25–30 overweight, >30 obese). Panel **b** and **c** percentage of patients displaying normal bone, osteopenia and osteoporosis, according to Bone Mineral Density values lumbar Tscore (panel **b**) and femoral T score (panel **c**), in BMI groups (<25 normal weight, 25–30 overweight, >30 obese)

Figure 4 Panel a shows the mean FMBD and LBMD as Tscore and Z-score in three BMI group: normal (BMI < 25), overweight (BMI: 25–30) or obese (BMI > 30).Only in normal BMI group the mean lumbar T-score and femoral Z-score revealed a decrease of BMD but since the BMD values of the group are quite heterogeneous, Standard Deviation resulting in quite high. The percentage of normal bone, osteopenic and osteoporotic subject, according to lumbar (panel B) and femoral (panel C) T-score values, was evaluated into each BMI group. At the increase of BMI correspond a decrease of osteopenia and osteoporosis, and an increase of normal bone. In particular, according to femoral T-score, at BMI values over 30 there is a complete absence of osteoporotic subjects. These results indicates a decrease of osteopenia and osteoporosis according to BMI. Increase

### sRAGE and Adipokines

**Fig. 5:**
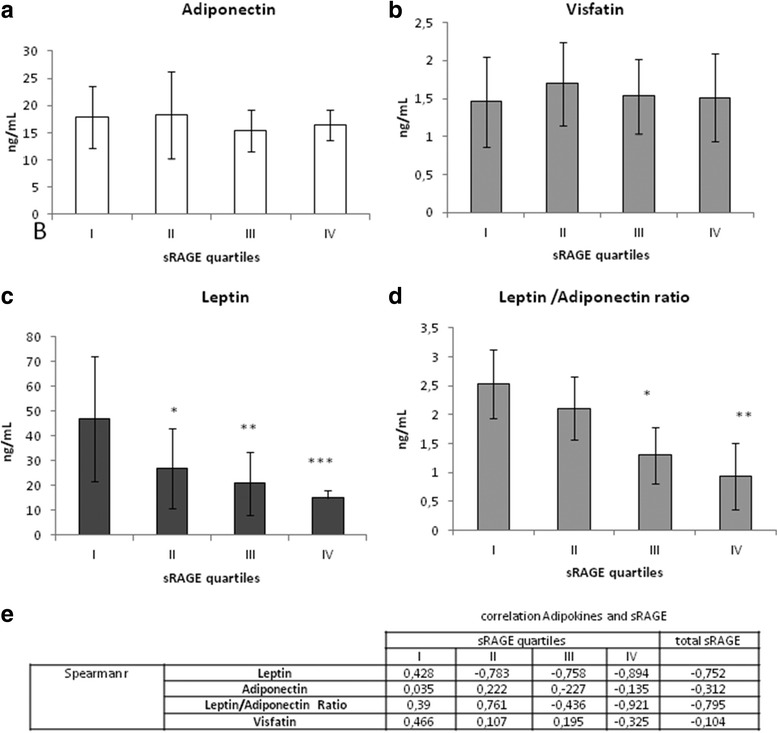
sRAGE and adipokines. Panel **a** Adiponectin (ng/mL) in patients in each sRAGE quartiles. Panel **b** Visfatin (ng/mL) in patients in each sRAGE quartiles. Panel **c** Leptin (ng/mL) in patients in each sRAGE quartile. Panel **d** Leptin /Adiponectin ratio in patients in each sRAGE quartiles. Panel **e** correlation (Spearmann) between Adipokines and sRAGE

Adiponectin and Visfatin (Fig. 5 panel a and b) displayed no significative differences among sRAGE quartiles, while Leptin and, as a consequence, Leptin/Adiponectin Ratio (Fig. 5 panel c and d) displayed a statistically significative decrease according to sRAGE quartiles.

Adiponectin and Visfatin display no significative correlation with total sRAGE or sRAGE quartiles, while Leptin and Leptin/Adiponectin Ratio displayed a good negative correlation (*p* < 0.001) with total sRAGE. This negative correlation increase according to sRAGE quartiles and it is particularly evident and significative (*p* < 0.001) n the forth quartile of sRAGE (panel E). These results indicates a negative correlation of sRAGE with leptin and Adiponectin.

## Discussion

Several evidences indicated that AGEs are involved in the pathogenesis of osteoporosis [5] and AGEs-RAGE interaction modulates osteoclasts and osteoblasts activity [24, 25]. For this reason we aimed to investigate the potential diagnostic role of soluble RAGE in osteoporosis. Our result clearly indicates that circulating level of soluble RAGE correlate with osteopenia and osteoporosis level (Fig. 1). This correlation is particularly evident by scoring RAGE into four quartiles.. These results clearly indicate that sRAGE correlates with disease progression from osteopenic to osteoporosis level. Usually sRAGE is reported to be high in healthy patients and lower in pathological condition, but this is not the first case where sRAGE, on the contrary, correlates with disease progression, behaving as a marker of disease progression. Indeed very recent evidenced that sRAGE, as widely described by Kailash Prasad [26], is not always a marker of good prognosis, because it can be reduced in some disease [27–30] and elevated in others [30–33]. The biological function of sRAGE is to bind circulating AGE, which could have detrimental effect on tissues, thus playing a protective role. For this reason in most of the cases, such as cardiovascular disorders, sRAGE, as a protective factor, is elevated in healthy subject and low in disease. On the contrary, in some cases such as diabetes renal disease, sRAGE is elevated compared to healthy controls as well as AGEs [5]. Similarly, recent evidences indicates a high level of sRAGE is present in acute respiratory distress and bronchiolitis [34], idiopathic pulmonary fibrosis [35] and it can correlate with disease severity in lung transplantation [35] and long term hemodyalisis patients [36].

It is known that AGEs accumulates in bone tissue at increasing ages, because bone tissue is susceptible to aGE accumulation due to its turnover. During bone remodelling bone cells are in close proximity to to AGE-modified proteins, such as collagen Type I and its degradation products. Bone cells express RAGE and can directly interact with Age, regulating differentiation maturation and function. Therefore, protein glycation in clearly involved in age- regulating bone disorders [37]. Franke et al. reported that on the one hand AGE-RAGE binding activates inflammatory response by inducing NFkB and TNF-α, on the other hand NFkB upregulates RAGE expression, thereby activating a self-promoting cicle among RAGE, Age and inflammatory mediators [37]. AGEs induced a decrease of osteoblast proliferation and osteoblast apoptosis [38]. In addition circulating AGE are reported to decrease bone strength [39]. The circulating sRAGE is the direct effect of the proteolitic cleavage of RAGE by metalloproteinases, and recent evidences suggested that diseases characterized by high level of MMPs, such as diabetes and renal disease [40, 41] display high level of serum sRAGE compared to healthy controls. Osteoporosis is characterized by a high expression of MMPs, acting on bone matrix resorption, and this can be the reason of high level of serum sRAGE in osteoporosis level. Therefore, circulating levels of sRAGE can be considered a direct indicator of circulating AGEs and, therefore, bone resorption status.

Consistently with this, our data indicates that serum sRAGE correlates with bone density decrease, ranging from the level of osteopenia to marked osteoporosis, confirming a correlation of serum sRAGE with the stage of bone fragility. This is also confirmed by the correlation of sRAGE quartiles with a strong marker of bone fracture risk, FGF23, as shown in Fig. 2. Being an important regulator of vitamin D and phosphorus, a key process in bone matrix turnover, Fibroblast growth factor-23 (FGF23) is considered a strong marker of bone fracture [42, 43]. In our patients there if we consider the whole population there is not a strong correlation of FGF23 and total fracture risk, as reported by Montalcini et Al, 2015 [23]. If we consider the classification according to T-score into normal bone, osteopenic and osteoporotic patients, a slightly increasing concentration of FGF23 results in osteopenic and osteoporotic group. In particular the only significative difference is observed in osteoporotic patients, displaying higher bone fragility, while at the level of osteopenia the risk of fracture is still too low to induce a marked and significative increase of FGF23. In consistence with that, there is a strong significative increase of FGF23 according to sRAGE quartiles, with a significative increase in particular in the third and even more in the highest quartile of sRAGE, suggesting a correlation of sRAGE with the increasing risk of bone fracture. This is also confirmed by the correlation analysis between serum FGF23 and sRAGE quartiles: even if the correlation with total sRAGE is not so strong, considering sRAGE quartiles classification a strong positive correlation emerges in the third and fourth quartiles. All these results clearly indicate that sRAGE can be considered a new biomarker of fracture risk in the osteoporosis.

It is well established that bone fracture risk is influenced not only by bone density but also by several metabolic factors, such as BMI and lipid metabolism [21, 22]. For this reason, we evaluated the association of BMI with the level of serum sRAGE. We observed a decrease of BMI according to sRAGE quartiles, and in particular if we categorize the patients into each sRAGE quartile according to the BMI standard classification (BMI < 25: normal weight, BMI 25–30 overweight, BMI > 30 obese) we observe a sticking prevalence of normal weight subject in the highest quartile of sRAGE. Accordingly, no obese subject is present in the highest quartile of sRAGE while the highest prevalence of obese subject results in the lowest quartile of sRAGE. This data indicate that there is a negative correlation with sRAGE and BMI.

In order to evaluate the relationship between BMI and bone status in our patients, we analyzed bone density score (lumbar and femora T-and Z-score) in the three BMI categories, as shown in Fig. 4. This result indicate that a lower bone density correspond to a lower BMI. This could appear contradictory, because, since BMI and lipid metabolism influences bone turnover, it could be supposed that elevated BMI and lipid metabolism correspond to a low bone density. However, it is well established that in obese patients there a counterbalancing mechanism: high body weight requires a stronger bone and, therefore, a higher bone density. Therefore, high BMI level, as confirmed in our patients, correspond to a higher bone density. This result on the one hand confirmed the positive correlation between BMI and bone density already reported in literature [44–46], on the other hand, given the negative correlation between sRAGE and BMI, confirm the role of sRAGE as a marker of bone fragility and fracture risk. In order to deeply correlate the relationship between s RAGE and lipid metabolism me evaluated a panel of the main adipokine (adiponectin, visfatin and leptin) and correlated them to serum sRAGE. According to s RAGE quartile, no significative differences are observed for Adiponectin and Visfatin. These results confirm previous evidences indicating that adiponectin and visfatin are controversial in osteoporotic patient s and cannot be considered good markers of osteoporosis [47, 48]. On the contrary, leptin displayed a statistically significative decrease into sRAGE quartiles, in accordance with BMI data. In addition to the single adipokines, the leptin/adiponectin ratio (LAR) is usually used to analyzed the lipid metabolism [49] and in our patients LAR displays the same trend of decrease, with significative lower level in the highest sRAGE quartile. This result are confirmed by correlation analysis, indicating that leptin and leptin/adiponectin ratio display a strong correlation with sRAGE, both as total sRAGE and into quartiles, in particular in the highest sRAGE quartile.

Taken together these results indicates that s RAGE display a strong negative correlation with leptin and LAR ratio.

This is the first study to our knowledge evaluating serum sRAGE in normal, osteopenic and osteoporotic patients. The limit of the study is to evaluate only few of the great number of parameter in the global evaluation of bone and lipid metabolism in fracture risk. This is pilot study evaluating the association of sRAGE and bone fragility in osteoporosis, and further investigation could be performed on a wider panel of parameter. The study is performed on a selected population of a small number of patients, all belonging to female gender and recruited into a local area, but since osteoporosis affects also male, further investigation could be performed on both genders, in order to replicate and extend the analysis.

This marker resulted clearly associated on the one hand to bone fragility and, on the other hand, with BMI and leptin. Since the ultimate goal of osteoporosis diagnosis and monitoring is the evaluation of fracture risk, it is important to take into account all the aspect affecting bone fragility. In this context sRAGE is particularly informative because serum sRAGE is able to provide, as a single marker, information about both the aspects of osteoporotic disease, represented by bone fragility and lipid metabolism.

The exact mechanism connecting AGEs and bone fragility is still unclear, but the level of advanced glycation end products (AGEs) have been recently reported to be inversely correlated with bone toughness and rigidity, due to their role in the inhibition of the synthesis of type I collagen, [39, 50, 51]. In addition, it is known that obesity is connected with increased amounts of AGE in the body [52, 53], leading to decreased bone toughness. In this context, our results shows that sRAGE would play a role in regulating AGEs, also according to lipid metabolism.

## Conclusion

The measure serum level of sRAGE could have a potential diagnostic role in the monitoring of osteoporosis progression, in particular in the evaluation of fracture risk, starting from the prevention and screening stage, to the osteopenic level to osteoporosis.
